# Paraneoplastic Glomerulonephropathy Associated With Renal Cell Carcinoma: A Descriptive Analysis of Published Reports

**DOI:** 10.7759/cureus.36928

**Published:** 2023-03-30

**Authors:** Xiaojie Zhang, Arushi Khurana, Samina Hirani, Jason Kidd, Asit Paul

**Affiliations:** 1 Department of Hematology and Oncology, University of California San Diego, San Diego, USA; 2 Division of Hematology, Mayo Clinic, Rochester, USA; 3 Department of Hematology, Oncology and Palliative Care, Virginia Commonwealth University School of Medicine, Richmond, USA; 4 Department of Nephrology, Virginia Commonwealth University School of Medicine, Richmond, USA; 5 Massey Cancer Center, Virginia Commonwealth University School of Medicine, Richmond, USA; 6 Department of Hematology, Oncology, and Palliative Care, Virginia Commonwealth University School of Medicine, Richmond, USA

**Keywords:** membranoproliferative nephropathy, iga nephropathy, minimal change disease, membranous nephropathy, paraneoplastic glomerulopathy, renal cell carcinoma

## Abstract

Paraneoplastic glomerulonephropathy (PGN) is a rare paraneoplastic syndrome that is associated with a variety of malignancies. Patients with renal cell carcinomas (RCCs) often develop paraneoplastic syndromes including PGN. To date, objective diagnostic criteria of PGN are not defined. As a result, the true occurrences are unknown. Many RCC patients develop renal insufficiency in the course of their disease, and diagnosis of PGN in this population is challenging and often delayed, which may lead to significant morbidity and mortality.

Here, we provide a descriptive analysis of the clinical presentation, treatment, and outcomes of 35 published patient cases of PGN associated with RCCs over the past four decades in PubMed-indexed journals. Most patients with PGN were male (77%), over 60 years of age (60%), and diagnosed with PGN prior to or concurrent with their diagnosis of RCC (20% prior, 71% concurrent). Membranous nephropathy (34%) was the most common pathologic subtype. Among the patients with localized RCCs, 16 (67%) of 24 patients had improvement in PGN compared to 4 (36%) of 11 patients with metastatic RCCs. All 24 patients with localized RCCs underwent nephrectomy, but patients who were treated with nephrectomy with immunosuppression (7/9, 78%) had a better outcome than patients who were treated with nephrectomy alone (9/15, 60%). Among the patients with metastatic RCCs, patients who were treated with systemic therapy along with immunosuppression (4/5, 80%) had a better outcome than those who were treated with systemic therapy, nephrectomy, or immunosuppression alone (1/6, 17%).

Our analysis demonstrates the importance of cancer-specific therapy; nephrectomy in localized disease and systemic therapy in metastatic disease, along with immunosuppression, was the effective management of PGN. Immunosuppression alone is not adequate in most patients. This is distinct from other glomerulonephropathy and warrants further study.

## Introduction and background

Glomerulonephropathy (GN), in the form of nephrotic syndrome, nephritic syndrome, or unexplained renal insufficiency, can be part of a paraneoplastic syndrome associated with malignancy. The first case, so-called paraneoplastic glomerulonephropathy (PGN), was described in 1922 by Galloway in association with Hodgkin’s disease [1,2]. Several reports since then have been published describing patients with solid tumors and hematologic malignancies. Common solid tumors reported to be associated with PGN include lung and gastrointestinal adenocarcinomas, as well as urologic malignancies [3,4].

PGN can be the initial presentation of renal cell carcinoma (RCC) or a sign of recurrence. Diagnosis is primarily clinical and is made based on a temporal relationship and the exclusion of other causes. As a result, true occurrences are unknown. Timely recognition of PGN is crucial for the effective management of both the underlying malignancy and associated paraneoplastic complications. However, PGN poses a diagnostic challenge given the lack of unifying criteria defining the syndrome and the confounding side effects of cancer treatment. Both nephrectomy and systemic therapies for RCCs can potentially cause renal insufficiency, thus obscuring the diagnosis. In addition, PGN may be challenging to differentiate from idiopathic and other forms of nephropathies because of overlapping clinical features. This distinction is important since corticosteroids and other immunosuppressive agents are effective in idiopathic and immune-related nephritis, but their role in PGN is uncertain.

To date, there have not been published prospective studies, retrospective analyses, or systematic reviews of PGN in patients with RCCs. In this article, we conducted a review of published cases of PGNs associated with RCCs over the last four decades in PubMed-indexed journals and report their clinical presentations, treatment, and outcomes.

This article was previously presented as a meeting abstract poster at the American Association of Cancer Research Annual Meeting 2020 (Abstract #3527).

## Review

Methods

We conducted a search to identify published reports on RCC-associated PGN in English language in PubMed-indexed journals between January 1, 1980, and March 30, 2021. Keywords used were “RCC,” “renal cell carcinoma,” “paraneoplastic glomerulopathy,” membranous nephropathy,” “membranoproliferative,”, nephropathy,” “minimal change disease,” “crescentic,” and “IgA Nephropathy.” Our inclusion criteria were as follows: adult patients with biopsy-proven nephrotic syndrome or nephritic syndrome diagnosed before, after, or around the same time as a biopsy-proven RCC. Our exclusion criteria included a history of documented autoimmune disease or rheumatological disease, non-RCC solid tumors or hematologic malignancies, and renal insufficiency secondary to exposure to nephrotoxic agents.

We collected data regarding the basic demographics of the patients, including age, gender, RCC histology and stage, and GN subtype, as well as the temporal relationship between the diagnosis of GN and the diagnosis of RCC. We also extracted data regarding the types of interventions received by the patients, including nephrectomy (partial vs total nephrectomy), immunosuppression, systemic therapy, and/or other medical management. Immunosuppression included treatments such as glucocorticoids (intravenous or oral), azathioprine, and cyclophosphamide. Systemic therapies included immunotherapy, targeted therapy, and chemotherapy. Other medical management included any pharmacologic agents outside of the aforementioned categories, such as angiotensin-converting enzyme inhibitors and diuretics. The response to interventions received by patients with PGN was also recorded, including improvement, hemodialysis (HD) requirement, death, and unspecified. Clinical improvement was defined as an improvement or resolution of nephrotic or nephritic syndrome supported by reduced proteinuria, improvement in creatinine clearance or glomerular filtration rate, and/or clinical improvement in edema.

Result

**Figure 1:**
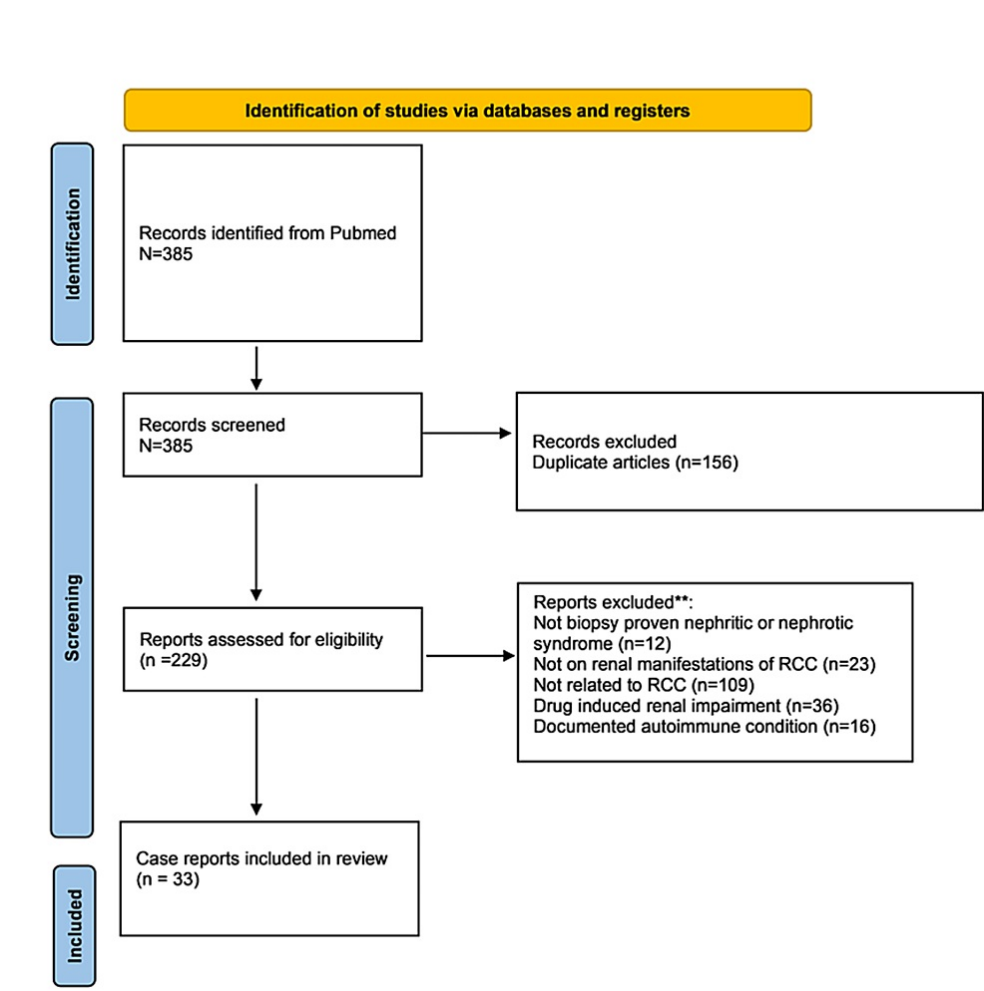
PRISMA flowchart PRISMA, Preferred Reporting Items for Systematic Reviews and Meta-Analyses

A total of 385 published papers were identified in PubMed-indexed journals, which included the search words during the specified time period. Among those, 32 case reports, which included 32 unique patient cases, and one case series, which included three unique patient cases, fulfilled the inclusion criteria. The selection process of articles is shown in the PRISMA flowchart in Figure 1 [5].

Individual patient data, including age, sex, and histological subtype, are described in Table 1. The majority of the patients were male (77%) and older (median age of 64 years; range: 26 to 78 years). PGNs were diagnosed concomitantly with the diagnosis of RCC in most patients (71.4%). Membranous nephropathy (MN) was the most frequent subtype of GN (34%). The leading histological subtype of RCC associated with PGN was clear cell RCC (49%) followed by papillary RCC, oncocytoma, and sarcomatoid RCC. In approximately one-third of patients, RCC subtype was not reported. Treatment and clinical outcomes are summarized in Table 2.

**Table 1:** Baseline characteristics of patients Total number of patients=35 IgA, immunoglobulin A

Demographics	Number (%)
Age	
<50 years	5 (14.3)
50-60 years	9 (25.7)
>60 years	21 (60)
Sex	
Male	27 (77)
Female	8 (23)
Temporal relationship	
Prior to tumor detection	7 (20)
Concurrent with tumor detection	25 (71.4)
After tumor detection	3 (8.6)
Glomerulonephropathy	
Membranous nephropathy	12 (34.1)
Minimal change disease	6 (17.1)
Focal segmental glomerulonephritis	5 (14.3)
IgA nephropathy	5 (14.3)
Crescentic glomerulonephritis	5 (14.3)
Membranoproliferative glomerulonephritis	1 (2.9)
Unspecified	1 (2.9)
Histological subtype	
Clear cell	17 (48.6)
Papillary	3 (8.6)
Chromophobe cell	0 (0)
Medullary	0 (0)
Sarcomatoid	1 (2.9)
Oncocytoma	2 (5.7)
Unspecified	12 (34.3)
Tumor extent	
Localized	24 (68.6)
Metastatic	11 (31.4)

**Table 2:** Outcome of treatment ^a^Five cases of partial nephrectomy, eight cases of total nephrectomy, two unspecified cases. ^b^One case of partial nephrectomy, two cases of total nephrectomy, and six unspecified cases. ^c^One case of total nephrectomy. ^d^One case of immunotherapy. ^e^One case of chemotherapy, three cases of targeted therapy, one case of immunotherapy. ^f^Two cases of unspecified, two cases of total nephrectomy. ^g^Two cases of supportive care.

Stage of RCC	Treatment	Total Number	Improved (%)	HD dependence (%)	Death	Unknown (%)
Non-metastatic		24	16 (%)	4 (%)	1 (%)	3 (%)
	Nephrectomy^a^	15	9	3	1	2
	Nephrectomy^b^ + immunosuppression	9	7	1	0	1
Metastatic		11	4 (%)	1 (%)	6 (%)	0 (%)
	Nephrectomy^c^	1	0	0	1	0
	Immunosuppression^d^	2	0	0	2	0
	Systemic therapy^e^ and nephrectomy	1	0	0	1	0
	Systemic therapy^f^ + immunosuppression	5	4	0	1	0
	Others^g^	2	0	1	1	0

Overall, 24 patients had localized disease and 11 patients had metastatic disease. Among the patients with localized RCCs, 67% (16 of 24) had improvement in PGN compared to 36% (four of 11) patients with metastatic RCC. All 24 patients with localized RCC underwent nephrectomy, but patients who were treated with nephrectomy with immunosuppression (7/9, 78%) had a better outcome than patients who were treated with nephrectomy alone (9/15, 60%). Among the patients with metastatic RCC, patients who were treated with systemic therapy along with immunosuppression (4/5, 80%) had a better outcome than those who were treated with systemic therapy, nephrectomy, or immunosuppression alone (1/6, 17%). In the latter group, none of the patients who received nephrectomy, systemic therapy, or immunosuppression alone had an improvement in PGN. These results showed that cancer-targeted therapy, nephrectomy in localized RCC, and systemic in metastatic disease, along with immunosuppression yielded better outcomes, i.e., improvement in PGN, decreased need for hemodialysis, and survival.

Discussion

We identified only 35 patients who had a diagnosis of nephrotic or nephritic syndrome associated with a diagnosis of RCC fitting the clinical criteria of PGN over four decades (Table 3). This highlights the rarity of PGNs associated with RCCs in the published literature. In a case series reporting PGN in solid tumors, the estimated prevalence was 10-20% because of the application of more liberal diagnostic criteria and autopsy-based analysis. The diagnosis of PGN is often based on a temporal association of GN with a malignancy (either before or after diagnosis), the absence of an alternate etiology, improvement with treatment of malignancy, and worsening of the condition with a recurrence or increase in the burden of malignancy [6]. These criteria are subjective and may or may not always reflect the true incidence of the condition. In a real-world scenario, a coincidental diagnosis of a GN with RCC can potentially be overdiagnosed as a PGN. For example, MN is the most common type of PGN associated with solid tumors and is difficult to differentiate from idiopathic MN. A clinically available biomarker can improve the diagnostic accuracy of PGN. In certain tumors, it is possible to establish a direct association of a GN with the malignancy. An example includes the detection of carcinoembryonic antigens in patients with colon cancer and antitumor antibodies within the glomerular immune deposits [4]. This is difficult in RCCs because of the lack of clinically acceptable and measurable tumor antigens. Further improvement in the diagnosis is possible by using newer immunoassays and identification of biomarkers of the disease. Immunoassays to detect PLA2R1 antibodies for the diagnosis of idiopathic MN are reported to have a sensitivity and specificity of 70% and 90% [7], respectively, and their absence supports the diagnosis of cancer-associated MN. In the absence of a widely available diagnostic tool, a high degree of clinical suspicion continues to be paramount in the diagnosis and management of PGN.

**Table 3:** Individual patient’s demography, treatment, and outcome CGN, chronic glomerulonephritis; FSGN, focal segmental glomerulosclerosis; HD, hemodialysis; IgA, immunoglobulin A; MCD, minimal change disease; MGN, membranous glomerulonephritis; MPGN, membranoproliferative glomerulonephritis; PGN, paraneoplastic glomerulopathy

	Reference	Age	Gender	Histological subtype	Localized vs metastatic	PGN	Treatment	Outcome
1	Karim et al. [8]	68	F	Clear	Localized	CGN	Total nephrectomy, steroids	Unspecified
2	Nunez et al. [9]	55	M	Unspecified	Localized	MGN	Partial nephrectomy	Improved
3	Kapoulas et al. [10]	72	M	Clear	Localized	MGN	Total nephrectomy	HD
4	Forland et al. [11]	49	M	Oncocytoma	Localized	MCD	Nephrectomy, steroids	Improved
5	Norris et al. [12]	61	M	Clear	Localized	FSGN	Total nephrectomy	HD
6	Feriozzi et al. [13]	50	M	Oncocytoma	Localized	FSGN	Nephrectomy, cyclophosphamide, azathioprine, steroids	Stably reduced renal function
7	Abu-Romeh et al. [14]	50	M	Papillary	Localized	IgA	Total nephrectomy	Improved
8	Tanaka et al. [15]	61	M	Clear	Localized	IgA	Total nephrectomy	Improved
9	Mimura et al. [16]	66	M	Clear	Localized	IgA	Nephrectomy, steroids	HD
10	Mimura et al. [16].	58	M	Clear	Localized	IgA	Nephrectomy, steroids	Improved
11	Mimura et al. [16]	59	M	Clear	Localized	IgA	Nephrectomy	Improved
12	Yaghoubi et al. [17]	55	M	Papillary	Localized	Unspecified	Total nephrectomy	Unspecified
13	Abrich et al. [18]	75	F	Unspecified	Localized	CGN	Partial nephrectomy, systemic therapy, steroids	Improved
14	Fujita et al. [19]	62	M	Clear	Localized	MGN	Partial nephrectomy	Improved
15	Woodrow et al. [20]	64	M	unspecified	Localized	MCD	Nephrectomy, steroids	Improved
16	Auguet et al. [21]	78	M	Unspecified	Localized	MCD	Nephrectomy, steroids	Improved
17	Ahmed et al. [22]	65	M	Clear	Localized	MGN	Nephrectomy	Improved
18	Kagan et al. [23]	42	M	Clear	Localized	CGN	Partial nephrectomy	HD
19	Hazar et al. [24]	72	M	Clear	Localized	MGN	Partial nephrectomy	Improved
20	Togawa et al. [25]	57	M	Unspecified	Metastatic	MGN	Total nephrectomy	Died
21	Kuroda et al. [26]	77	M	Clear	Localized	MGN	Total nephrectomy	Improved
22	Sommer et al. [27]	41	M	Clear	Metastatic	FSGN	Total nephrectomy, steroids	HD, died
23	Devarsetty et al. [28]	65	M	Unspecified	Metastatic	MGN	ACE inhibitor	Progressed
24	Tucci et al. [29]	68	M	Sarcomatoid	Metastatic	FSGN	Total nephrectomy, systemic therapy, steroids	Improved
25	Nishibara et al. [30]	69	M	Clear	Metastatic	MGN	Steroids, diuretics	Died
26	Kai et al. [31]	59	F	Unspecified	Metastatic	CGN	Nephrectomy, immunotherapy	HD, died
27	Stein et al. [32]	76	F	Unspecified	Localized	MGN	Total nephrectomy	Died
28	Jain et al. [33]	35	F	Unspecified	Metastatic	CGN	None	Died
29	Dabrowski et al. [34]	58	M	Papillary	Localized	FSGN	Total nephrectomy	Unspecified
30	Song et al. [35]	69	M	Clear	Localized	MGN	Partial nephrectomy	Improved
31	Mogili et al. [36]	65	M	Unspecified	Localized	MPGN	Total nephrectomy, steroids	Improved
32	Yasuda et al. [37]	26	F	Unspecified	Metastatic	MGN	Nephrectomy, systemic therapy, steroids	Improved
33	Ihara et al. [38]	64	M	Clear	Metastatic	MCD	Nephrectomy, systemic therapy, steroids	Improved
34	Abouchacra et al. [39]	69	M	Unspecified	Metastatic	MCD	Steroids, cyclophosphamide	HD, died
35	Khurana et al. [40]	73	M	clear	Metastatic	MCD	Systemic therapy, surgery of the metastatic site, steroids	In remission

Our review reveals that an overwhelming majority of patients were male and older than 60 years. This is expected because RCC is primarily a disease of the elderly. It emphasizes the importance of a high degree of suspicion of associated malignancy in older patients with unexplained nephritic or nephrotic syndrome. Most patients had MN, which is the most common type of paraneoplastic nephropathy in other solid tumors. The majority of patients with RCC had clear cell RCC.

The management strategy of PGN is far from conclusive, and a practical, evidence-based approach is unknown. In real practice, immunosuppression in the form of corticosteroids is the mainstay of management. Albeit a small number of patients, our analysis showed that cancer-specific therapy, nephrectomy in localized RCC, or systemic anticancer therapy in metastatic RCC, along with immunosuppression led to the best outcome of PGN. The outcome was better compared to patients who were treated with cancer-specific therapy or immunosuppression alone.

## Conclusions

PGN associated with RCC was rare. We identified only 35 patients of RCC-associated PGN in the reports published over four decades. MN was the most common type of PGN associated with RCC. The results showed that patients who were treated with cancer-specific therapy combined with immunosuppression had a better outcome than those who were treated with nephrectomy, systemic therapy, or immunosuppression alone. Overall, these results support the concept that successful management of paraneoplastic syndrome requires definitive therapy or systemic therapy to treat the underlying malignancy.
